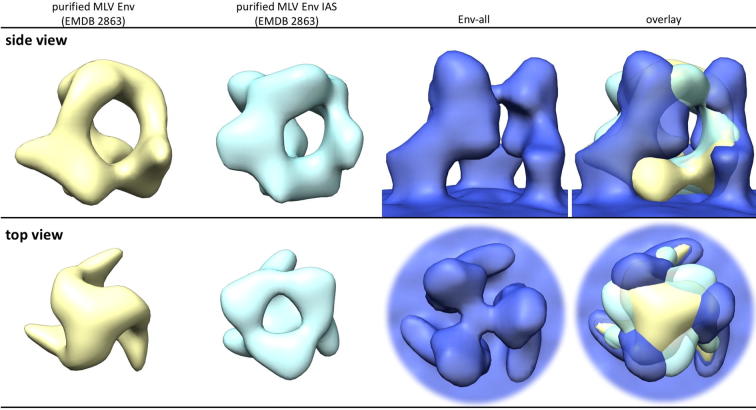# Native structure of a retroviral envelope protein and its conformational change upon interaction with the target cell

**DOI:** 10.1016/j.jsb.2016.06.017

**Published:** 2017-02

**Authors:** Christiane Riedel, Daven Vasishtan, C. Alistair Siebert, Cathy Whittle, Maik J. Lehmann, Walther Mothes, Kay Grünewald

**Affiliations:** a Division of Structural Biology, Wellcome Trust Centre for Human Genetics, University of Oxford, Oxford OX3 7BN, UK; b Institute of Virology, University of Veterinary Medicine, Vienna, Austria; c Department of Life Sciences and Engineering, University of Applied Sciences Bingen, Germany; d Department of Microbial Pathogenesis, Yale University School of Medicine, New Haven, CT, USA

**Keywords:** *Retroviridae*, Murine leukemia virus, Env, Virus entry, Electron cryo tomography, Sub-volume averaging

## Abstract

Enveloped viruses enter their host cells by membrane fusion. The process of attachment and fusion in retroviruses is mediated by a single viral envelope glycoprotein (Env). Conformational changes of Env in the course of fusion are a focus of intense studies. Here we provide further insight into the changes occurring in retroviral Env during its initial interaction with the cell, employing murine leukemia virus (MLV) as model system. We first determined the structure of both natively membrane anchored MLV Env and MLV Env tagged with YFP in the proline rich region (PRR) by electron cryo tomography (cET) and sub-volume averaging. At a resolution of ∼20 Å, native MLV Env presents as a hollow trimer (height ∼85 Å, diameter ∼120 Å) composed of step-shaped protomers. The major difference to the YFP-tagged protein was in regions outside of the central trimer. Next, we focused on elucidating the changes in MLV Env upon interaction with a host cell. Virus interaction with the plasma membrane occurred over a large surface and Env clustering on the binding site was observed. Sub-volume averaging did yield a low-resolution structure of Env interacting with the cell, which had lost its threefold symmetry and was elongated by ∼35 Å in comparison to the unbound protein. This indicates a major rearrangement of Env upon host cell binding. At the site of virus interaction, the otherwise clearly defined bilayer structure of the host cell plasma membrane was much less evident, indicative of integral membrane protein accumulation and/or a change in membrane lipid composition.

## 1 Introduction

Murine leukemia viruses (MLV) are members of the Gammaretroviruses that cause leukemia and lymphoma in mice. Depending on their receptor usage, they are assigned to 3 classes; ecotropic, amphotropic or xenotropic. The viral particle diameter determined by electron cryo microscopy (cEM) varies from 100 ± 10 nm (Förster et al., 2005) to 124 ± 14 nm (Yeager et al., 1998) depending on the producer cell line. The viral envelope glycoprotein Env (∼74 kDa) is the only viral protein present in the virus membrane and is responsible for interaction with the cellular receptor mCAT (SLC7A1) in ecotropic viruses. Env is incorporated into the virus envelope as a trimer, with each protomer consisting of the surface unit (SU, gp70) linked by a disulfide bond to the transmembrane unit (TM, Pr15E) (Opstelten et al., 1998, Pinter et al., 1997). SU consists of three domains, the receptor binding domain (RBD), the proline rich region (PRR), and the C-terminal domain, and contains several glycosylation sites (Kayman et al., 1991, Pinter and Honnen, 1988). Insertion of protein sequences (including fluorophores like YFP) is only possible within the PRR, else loss of biological function occurs (Kayman et al., 1999a, Kayman et al., 1999b, Sherer et al., 2003). Interaction with the cellular receptor mCAT (Albritton et al., 1989), an integral membrane protein with 14 transmembrane domains, is suggested to occur via residues D86 (Bae et al., 1997, Davey et al., 1999, MacKrell et al., 1996), R85 and R97 (Bae et al., 1997) and W144 (Zavorotinskaya and Albritton, 1999) within the RBD of Friend MLV (FrMLV; another well-studied isolate is Moloney MLV [MoMLV], amino acid identity in Env >84%). On the receptor site, residues Asp232, Val233, Tyr235 and Glu237 have been determined as being essential for SU interaction (Albritton et al., 1993, Yoshimoto et al., 1993). These residues are located on extracellular loop 3 (residues 213–239).

The structure of the receptor binding domain of FrMLV Env (amino acid 1–236) has been determined by X-ray crystallography (Fass et al., 1997). The full-length structure in the viral membrane was determined by electron cryo tomography (cET) followed by sub-volume averaging (Förster et al., 2005) and after detergent extraction using cEM single particle analysis (Wu et al., 2008) from MoMLV produced in the mouse fibroblast cell lines CL-1 and MOV-3, respectively. Additionally, parts of the TM domain of MoMLV Env (Fass et al., 1996) and a xenotropic MLV (Aydin et al., 2014) have been crystallized in their post-fusion state, which contains a six helix bundle. Still, information about the changes that MLV Env undergoes upon interaction with the host cell is lacking. For HIV, indirect evidence supports the formation of an elongated pre-hairpin structure of TM upon initial contact with the cell membrane, which then folds back into the six-helix bundle to accomplish fusion (as reviewed in (Blumenthal et al., 2012)).

Here we present EM density maps of unbound MLV Env and its YFP-labeled form (Sherer et al., 2003) (Env-YFP) as generated by sub-volume averaging. Having determined the density maps of the unbound Env then allowed us to analyze the changes in Env upon binding of MLV to its host cell *in situ*, revealing major conformational changes.

## 2 Results

### 2.1 Structure determination of MLV Env and MLV Env-YFP

**Fig. 1:**
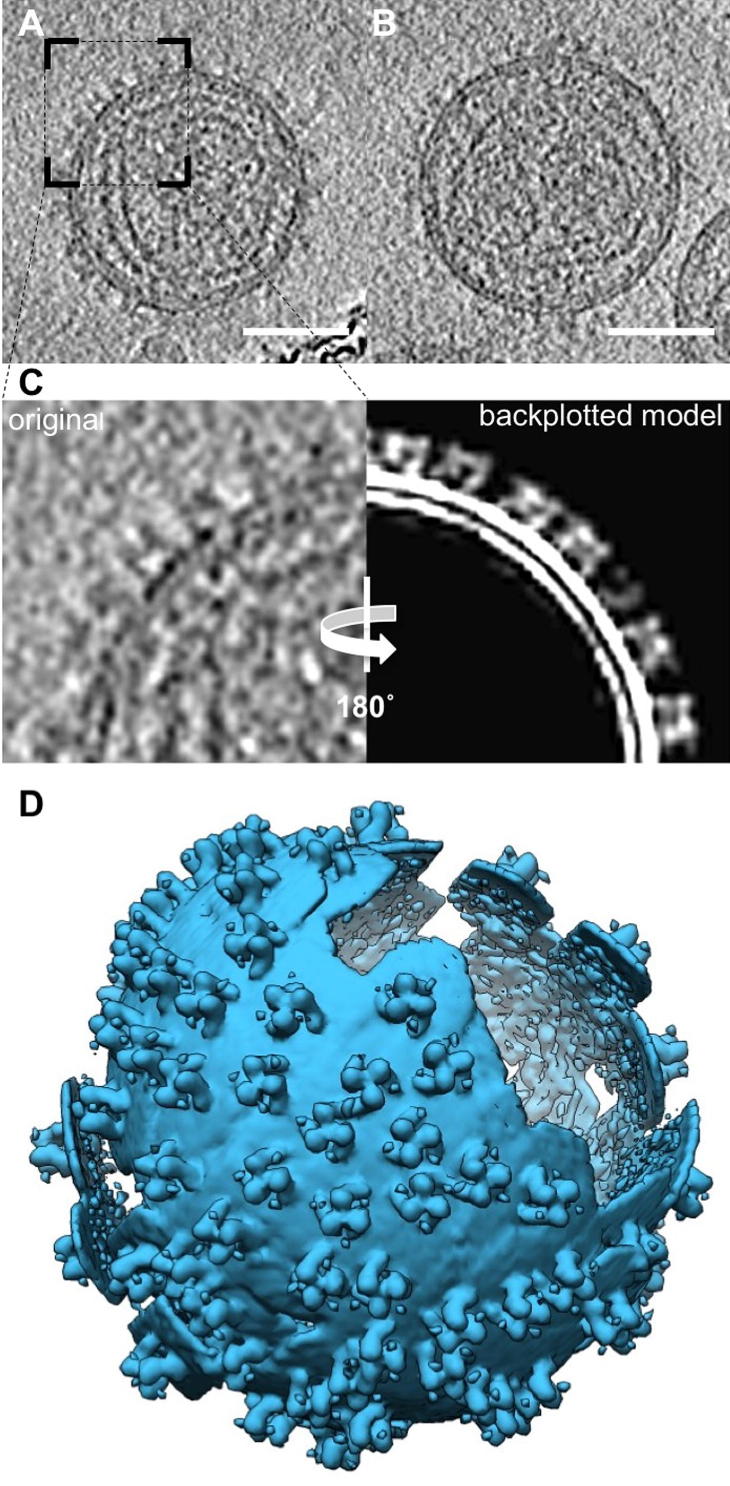
Electron cryo tomography and sub-volume averaging of Env. (A, B) Slice through a tomogram of COS1-derived virus like particles (VLPs) displaying Env (A) and Env-YFP (B). (C) Consensus between original data and sub-volume model. Left: detail of area marked in (A), and right: same area (vertically mirrored) showing the backplotted Env sub-volume average in the found positions and orientations. (D) Surface representation of the VLP shown in (A) generated by backplotting the Env average (as in (C), right). Scale bars: 50 nm.

Previously published structures of MLV Env differed quite significantly depending on the sample nature (isolated, detergent solubilized vs. natively anchored in membrane) and reconstruction technique used (cEM single particle vs. cET and sub-volume averaging) (Förster et al., 2005, Wu et al., 2008). In order to identify changes in MLV Env upon receptor interaction it was therefore necessary to generate an EM density map for the naïve, native membrane protein (i.e. the unbound state). We initially used COS-1 cell derived virus like particles (VLPs) carrying either Env or Env-YFP to determine the respective native structures in the membrane. We also included a replication competent FrMLV – labeled in the PRR with YFP and produced in DFJ8 cells – in the structure determination, as this virus was used to define the binding kinetics in fluorescence microscopy and subsequently for cell interaction experiments analyzed by cET. After reconstruction of the tomograms from the acquired tilt series (for details see Section 4), individual Env/Env-YFP particles were picked by hand to generate the initial dataset. Only fully Env/Env-YFP covered VLPs/viruses were included. Automated picking approaches, like local minima search (Zeev-Ben-Mordehai et al., 2014) or picking along predetermined lattices, were not successful. Representative VLPs carrying either Env or Env-YFP are depicted in Fig. 1A + B, respectively.

Differences in the morphology of Env versus Env-YFP can already be perceived in the raw tomograms, with Env-YFP densities being less clearly defined than Env densities. To generate 3D structures of Env and Env-YFP, reference-free sub-volume averaging was performed using the PEET software package (Nicastro et al., 2006). This process involves iteratively aligning and averaging all the macromolecules of interest (normally referred to as ‘particles’) based on the highest cross correlation coefficient (CCC) value against a reference, ultimately resulting in an electron density map with improved signal to noise ratio (SNR) and resolution. For the generation of the initial average, to be then used as a reference, all particles were oriented such that their Y-axes were approximately normal to the membrane. This was achieved by aligning the Y-axis of each particle to the vector between the center of the VLP/virus and the particle (Env) on the VLP/virus envelope. Early in the sub-volume averaging process (iteration 2 or 3, depending on the dataset), the structure exhibited clear threefold symmetry, with the symmetry axis being normal to the membrane. This is in accordance with the physiological trimeric state of Env. During subsequent iterations, the dataset was cleaned by removal of duplicates (criterion: ⩽92 Å distance between particle centers) and removal of particles that deviated by >15° from the initially assigned angle normal to the membrane.

To verify the validity of the Env particles and their orientation, the final average (EM density map) was plotted back on the original data, using the position and orientation determined for each individual particle (Fig. 1C, D). This validation helps judging the robustness of the procedure and used parameters, as well as the quality of the results, and can be performed in practice by toggling between the original data and the backplotted data. We found a good correlation of the individual Env trimes with the original data. Further, backplotting enabled us to analyze the relative orientations of the particles (Huiskonen et al., 2010, Maurer et al., 2013). No higher order symmetries in the lateral arrangement of Env on the membrane of the VLPs/viruses was detected.

### 2.2 MLV Env and Env-YFP are hollow trimers consisting of step-shaped protomers

**Fig. 2:**
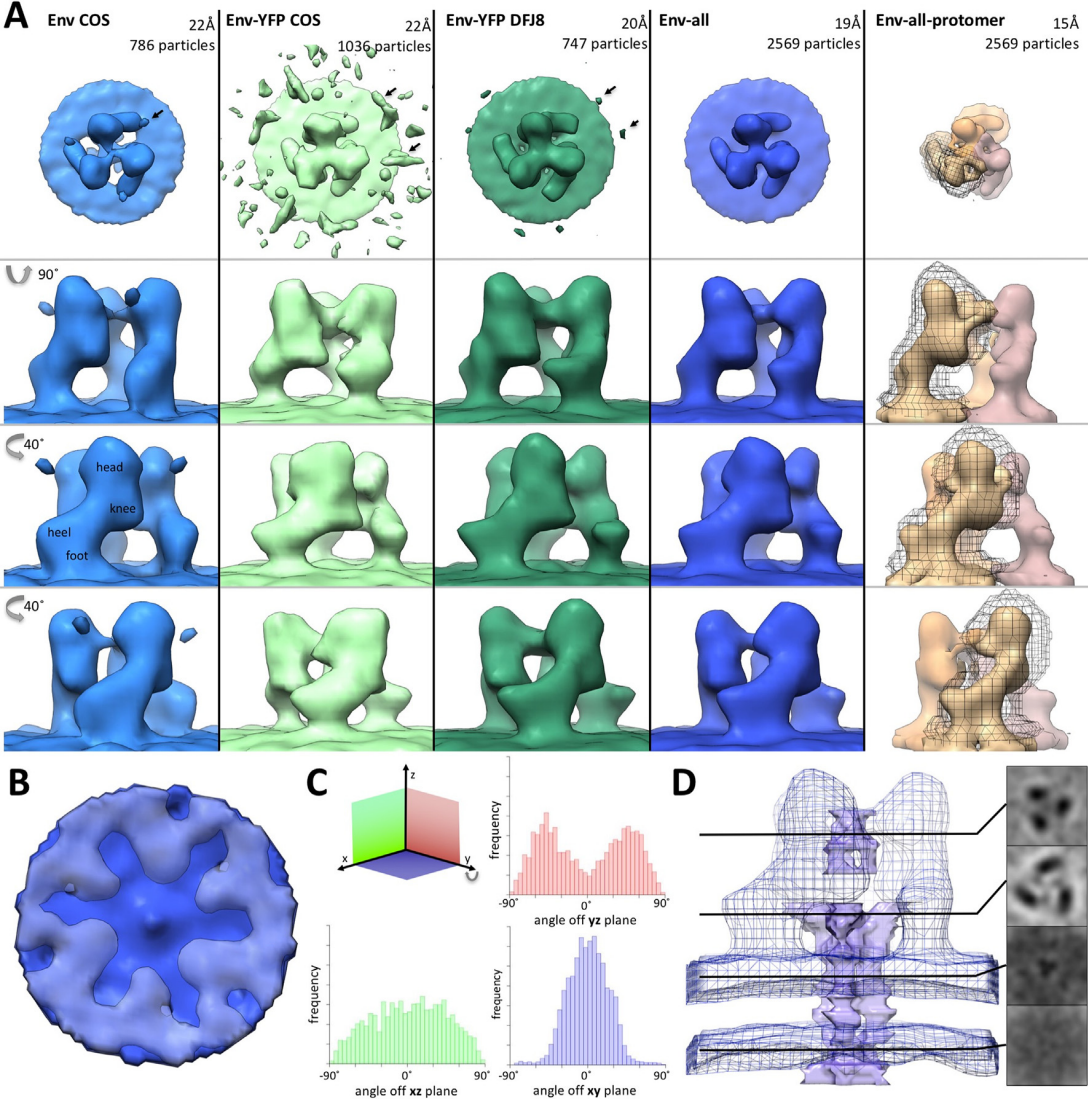
3D density maps of unbound Env and Env-YFP from sub-volume averaging. (A) Top and side views of the surface representations of maps (threshold based on estimated volume of 3× (SU + TM) excluding transmembrane and intraviral parts, see also Section 4) derived from VLPs carrying either Env (Env COS, colored light blue, EMD_3365) or Env-YFP (Env-YFP COS, colored light green, EMD_3363) as well as viruses carrying Env-YFP (Env-YFP DFJ8, colored dark green, EMD_3357). Also, the map for a combination of all three datasets (Env-all, colored dark blue) is depicted (Side and top view of Env-all, including viral envelope and capsid, as well distance measurements, are given in Supplementary Fig. 3). Additionally, the EM density map of the combined dataset, resulting from running the iterative alignment focused on a single protomer (using a tight mask in later iterations, EMD_3373), is shown (Env-all-protomer, protomers colored brown, salmon and orange, outline of one mask shown in black). The number of particles included in each reconstruction as well as the achieved resolution are given above each column. Arrows indicate densities outside the central trimer (see also Supplementary Fig. 1). In the side views of YFP-Env COS, YFP-Env DFJ8 and Env-all volumes <10,000 Å^3^ are not depicted. (B) Bottom view of the Env-all reconstruction, showing a central protrusion from the inner membrane leaflet and a lack of densities in the capsid layer (colored light grey-blue) underneath this protrusion. (C) Histograms visualizing the angular distributions of particles of all datasets depending on their angle relative to the xz-, yz- or xy-plane. (D) Lower threshold segmentation of densities present in the central cavity of the trimer (colored purple, based on Env-all which is shown as a blue outline). Note that these densities extend clearly throughout the viral envelope. Representative xy-slices of the electron density map are shown to the right.

**Supplementary Fig. 1:**
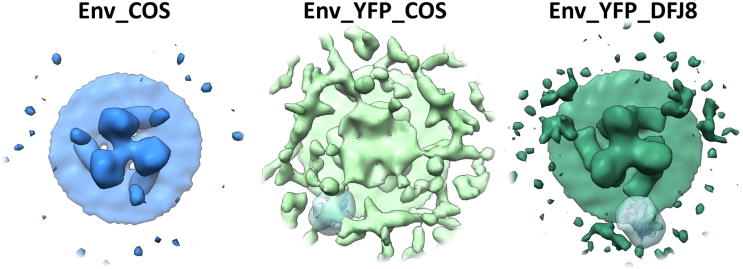


Our 3D reconstructions of MLV Env and Env-YFP consist of three step-shaped protomers, which we subdivided from membrane proximal to distal into foot, heel, knee and head sections (Fig. 2A). Smaller density differences between the Env and Env-YFP reconstructions occur in the heel and on the inside of the knee area, but they are not spacious enough to accommodate a YFP molecule within the central trimer. However, small densities outside the central trimer – designated arms – (indicated by black arrows in Fig. 2A), which are further away from the center of the trimer in Env-YFP, hint at an elongation of this structure due to the insertion of YFP, and this seems the most likely localization of parts of the very flexible PRR and, in case of Env-YFP, YFP. They are not large enough either to accommodate a YFP molecule, but might indicate the part of the YFP molecule that has the largest overlap between the different positions that YFP can accommodate due to its insertion into the PRR. Depiction of these densities at a lower threshold level (Supplementary Fig. 1) revealed more of this density and further support this possibility.

**Supplementary Fig. 2:**
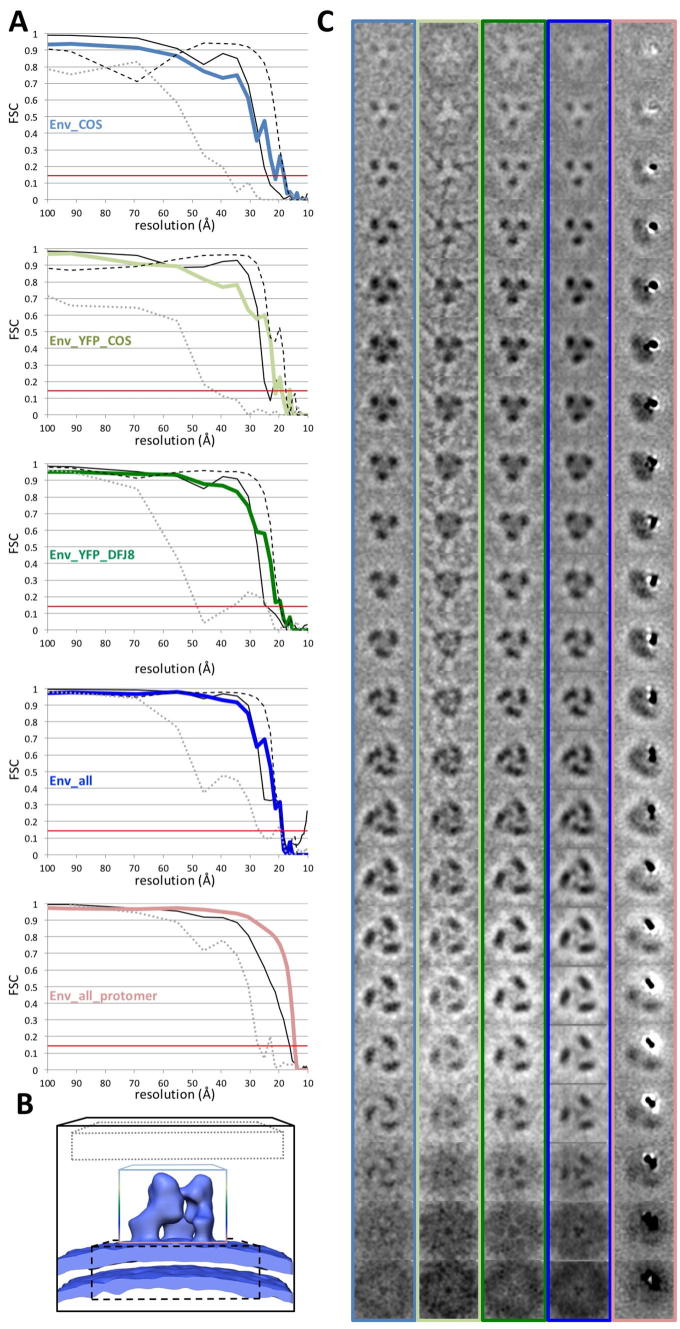


**Supplementary Fig. 4:**
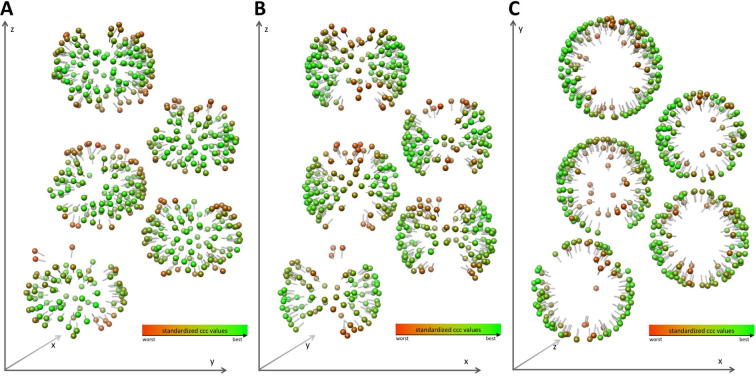


The resolution of these reconstructions was calculated using gold standard Fourier shell correlation (FSC) (Henderson et al., 2012) employing the 0.143 cutoff. Employing this approach, the final resolution of Env and of Env-YFP derived from COS-1 produced VLPs is 22 Å (from 786 and 1036 particles, respectively, particle numbers are given before application of threefold symmetry for all datasets), whereas it is 20 Å (from 747 particles) for Env-YFP reconstructed from DFJ8 derived FrMLV (shown together with xy slices of the density maps along the z-axis in Supplementary Fig. 2). Since YFP did not alter the shape of the central trimer, we combined all data sets, which resulted in a slightly increased resolution of 19 Å (Fig. 2A Env-all; from 2569 particles). Combining all data further allowed to test the homogeneity of the included particles by principle component analysis (Heumann et al., 2011). This did not unveil the presence of distinct classes of particles, but showed that the largest variability occurred in the head and heel area. Notably, these regions were also the most variable in the EM density maps of the split datasets used for FSC calculation. Only a minority of particles used in the reconstruction had an angle off the xy-plane of >45° (Fig. 2C) which corresponds to particles with a large z-component, e.g. top views. This under-representation might explain these observed variabilities, being most apparent in the top views of our reconstructions. Notably, the cross-correlation values for our reconstructions decreased with increasing z or y component (see pin models in Supplementary Fig. 4) consistent with a tomography missing wedge effect.

At the resolutions reached, the EM maps at the chosen contour levels did not exhibit any densities representing the TM domain of Env on the virus surface. This is consistent with previous studies of HIV Env showing that the TM densities are indiscernible at 15 Å resolution or lower (Bartesaghi et al., 2013). The most pronounced indication of TM protein density was a small central protrusion of the inner membrane leaflet (Fig. 2B, shown on Env-all). Interestingly, the capsid lattice position directly underlying this protrusion was of significantly lower density in all reconstructions, possibly indicative of a specific interaction of the TM C-terminus with the capsid layer. Applying a tight cylindrical mask in the subvolume averaging allowed the recovery of densities protruding from the inner membrane leaflet towards the capsid (data not shown).

However, when analyzing xy slices along the z-axis of the reconstructions – which emphasize features along the z-axis -, a continuous, albeit weak density was observed in the center of the trimer, which extended throughout both membrane leaflets. The result of a representative lower threshold segmentation of Env-all, placed in the respective EM density map at the higher threshold used before, is shown in Fig. 2D and might represent parts of TM.

**Fig. 3:**
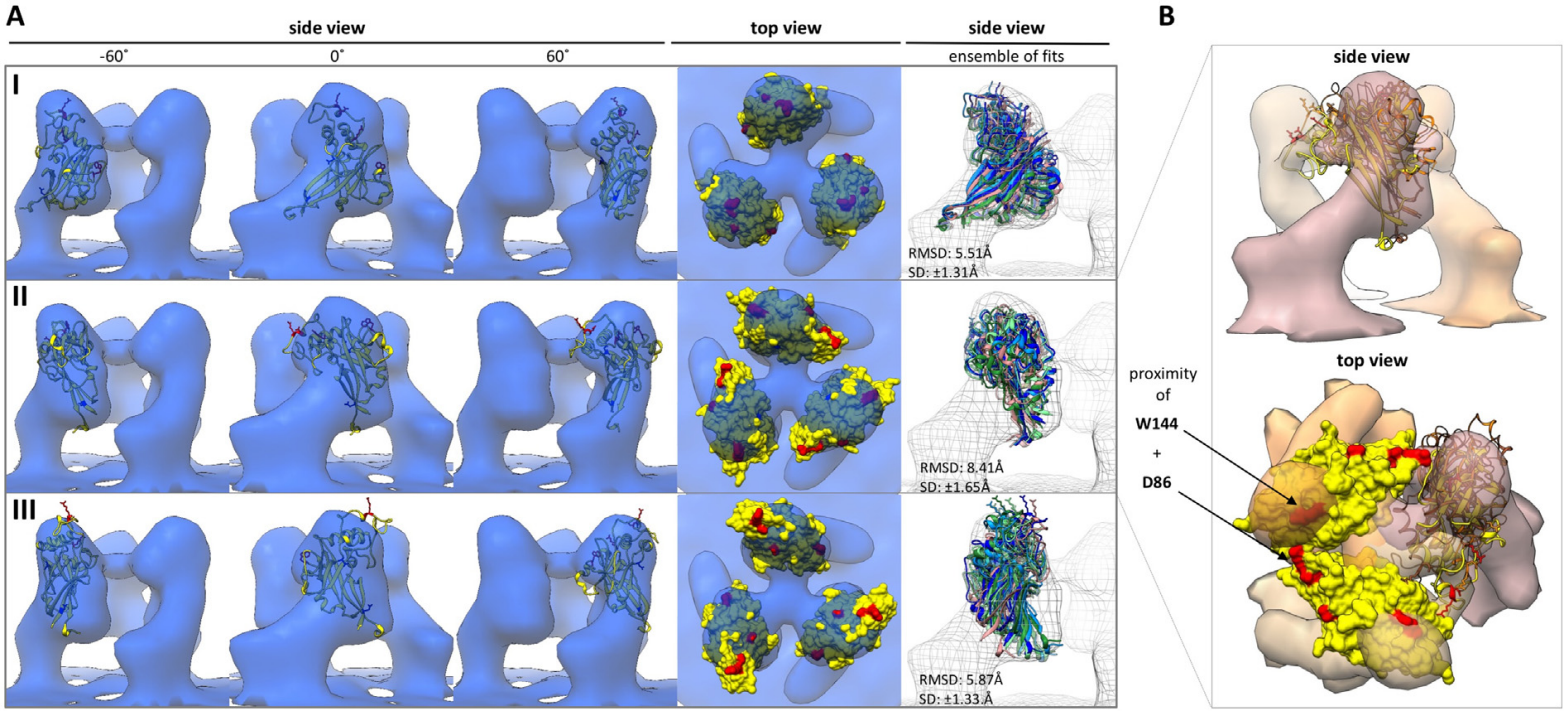
Env RBD fitting using a combined scoring system. (A) Fits I–III (from highest to lowest score) are shown in the corresponding Env-all density map in columns 1–4. On the RBD crystal structure (Fass et al., 1997) (shown in yellow) receptor interacting residues R85, D86, R97 and W144 are colored red, whereas glycosylation sites are colored blue. Column 5 gives an ensemble representing the variability of the fit in the different reconstructions (colored according to Fig. 2). (B) Fit II – the only one providing for close proximity of residues D86 and W144 – in the Env-all-protomer map is depicted in top and side view. Respective fits returned by a global search within the protomer are shown as orange and brown ribbons.

As there is the possibility that the protomers are not completely symmetrically aligned within the trimer due to conformational flexibility, and this being potentially limiting the attained resolution, a soft-edged mask was applied to each protomer and only this region was used for the alignment by sub-volume averaging. The result is shown in Fig. 2A (Env-all-protomer). This led to an improvement of the resolution for the single protomer to 15 Å (Env-all-protomer) (Supplementary Fig. 2). Still, docking of the crystal structure of the RBD (Fass et al., 1997) did not reveal a single best fit employing the global search option within UCSF Chimera (Pettersen et al., 2004). To evaluate the different possible fits of the rigid body fitting (optimized for either CCC or overlap), we employed a scoring scheme which included the CCC value, the overlap value, the number of hits in a global search as well as the number of receptor interacting residues exposed at the top of each of the used EM density maps (Env-COS [12 hits], Env-YFP COS [16 hits], Env-YFP DFJ8 [13 hits], Env-all [10 hits], Env-all-protomer [12 hits]). All values were determined in UCSF Chimera using the ‘fitmap’ command and standardized to allow score calculation. Once this score was calculated, only four fits scoring above average and being retrieved in all five EM density maps remained and were further evaluated. For this purpose, the fits were optimized in UCSF Chimera (again with the ‘fitmap’ command) in all EM density maps using a restrained global search (only allowing for small shifts and rotations), starting from the localization determined previously. Also, the RMSD of similar fits in different EM density maps was calculated and integrated into the score. Only fits with receptor interacting residues exposed in the head region of Env were included. The three fits that scored highest are depicted in Fig. 3, using Env-all as example. However, only one fit allowed for exposure of Trp 144 and D86 at the top of the map, with the two residues from two neighboring protomers being in closest proximity (Fig. 3) in the Env-all-protomer map.

### 2.3 Membrane distances of MLV Env-YFP interacting with host cells imply elongation of Env

**Fig. 4:**
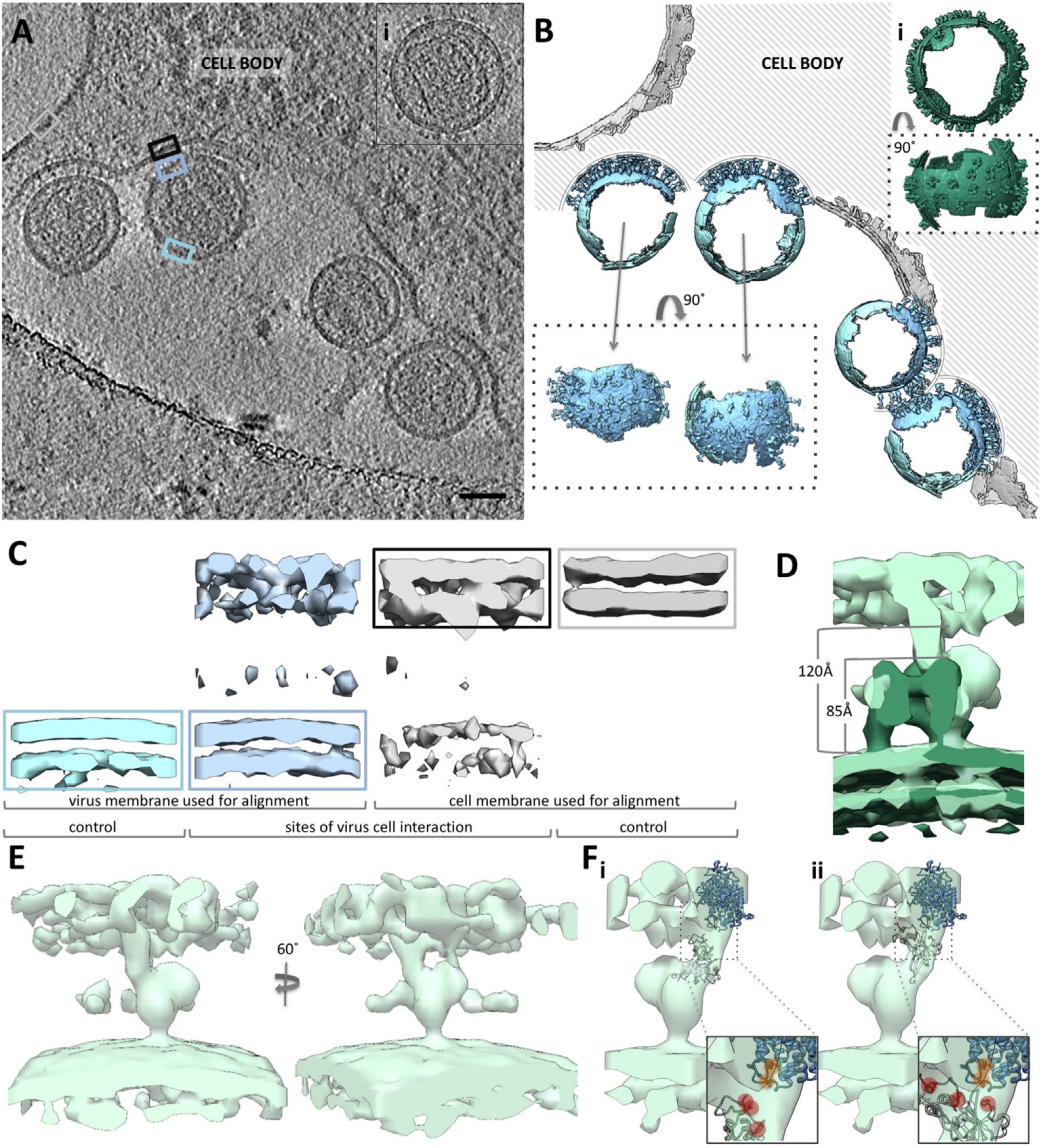
Env-YFP in interaction with the host cell. (A) Slice through a tomogram showing several viruses in contact with the cell. The particle shown in insert (i) is given as reference for the appearance of an unbound particle. Scale bar represents 50 nm. Color-outlined rectangular boxes indicate representative membrane positions used for the averages shown in (C). (B) Backplot of the final sub-volume averages onto (A) using the positions and orientations found for individual particles as part of the averaging. The color scheme follows the boxes in (A): interacting Env-YFP is depicted in light blue, non-interacting virus membrane in cyan and non-interacting cell membrane in grey. (i) Shows the same particle as in (A) as a backplot for comparison of Env-YFP density on the surface. (C) Central sections through sub-volume average derived maps at sites with or without interaction, generated either by alignment of cell membrane (grey) or virus membrane (light blue) only. The respective representative picking areas are indicated as color-outlined boxes in (A). (D) Overlay of unbound Env-YFP (dark green) and interacting Env-YFP (light green). Membrane distances and the height of unbound Env-YFP are indicated. (E) Surface representation of the EM density map of interacting Env-YFP filtered to 50 Å (represents a cutoff of 0.8 in non gold standard FSC; the data set did not provide for enough particles for gold standard analysis). (F) For scale, two different putative localizations of the RBD in the interacting Env-YFP EM density map are shown. RBD receptor interacting residues are colored red. Further, a putative structure of mCAT (generated using the Phyre2 server (Kelley et al., 2015)) has been added with the Env interacting residues shown in orange.

**Supplementary Fig. 5:**
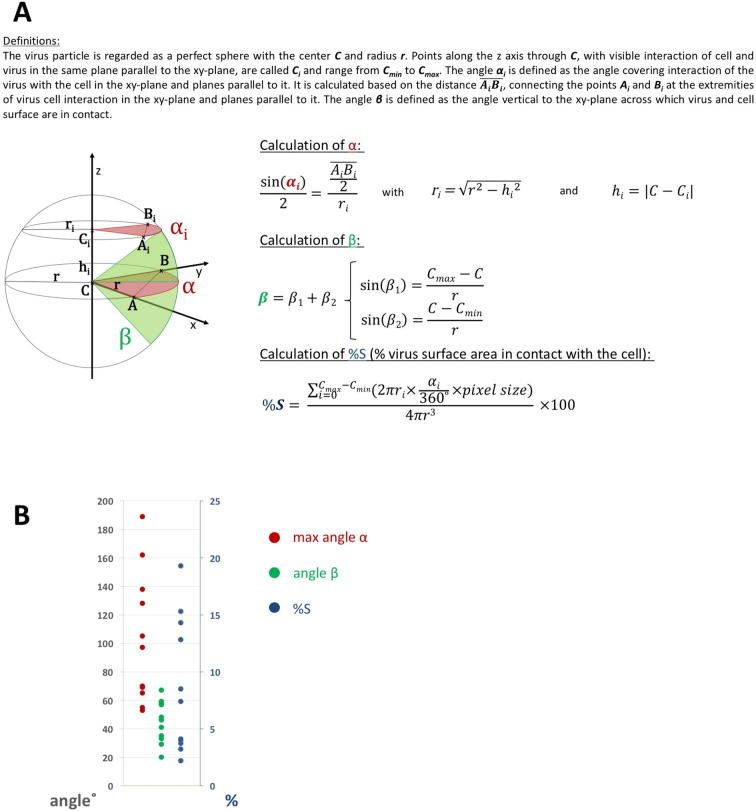


Retrovirus fusion has been studied in most detail for HIV and experimental evidence suggests the elongation of TM in a pre-hairpin conformation upon first contact with the cell membrane (reviewed in (Melikyan, 2014)). TM is subsequently folding back into a six-helix bundle to mediate membrane fusion. We allowed MLV Env-YFP viruses to bind to rat derived XC cells, which constitutively expresses mCAT labeled with CFP (Lehmann et al., 2005), at 37 °C in full medium (for more detail see Section 4). One to three minutes after virus addition, the cells were plunge frozen and analyzed by cEM. In areas where low magnification projection images showed viruses in close proximity to cells, cET tilt series were acquired. In the reconstructed 3D volumes, multiple events showing viruses in direct contact with the cell were found (Fig. 4A). Densities connecting the virus membrane with the cell membrane were clearly present in the acquired tomograms and plasma membrane curvature was observed in these areas. To characterize these interactions more closely, the maximum angle of the virus particle surface in contact with the cell was determined in the xy plane (α), and vertical to the xy plane (β) (Supplementary Fig. 5). Maximum values of α ranged between 53° and 189° (average 103°, n = 11), whereas values of β were distributed between 20° and 67° (average 45°, n = 11). Also, the virus surface area in contact with the cell was estimated and ranged between 2.2 and 19.3% of the virus surface (average 8.6%, n = 11, all calculations were based on the assumption that the virus is a perfect sphere). When larger virus surface areas were observed in contact with the cell, no actin filaments were present directly below the plasma membrane. These findings indicate the utilization of a large contact area with the cell during MLV entry if no restrictions by the cytoskeleton are encountered.

Notably, the distance between the viral and cellular membranes was fairly uniform, which made this dataset applicable for sub-volume averaging. Particles were picked manually and further processed for sub-volume averaging as described for the reconstruction of unbound Env. For comparison, Env free membrane regions on the virus and the plasma membrane were averaged too. A backplot of the resulting average maps on the original data is shown in Fig. 4B.

In comparison to unbound viruses, the local density of Env-YFP trimers present at the interaction site was increased, suggesting a clustering of Env upon binding. The average nearest neighbor distance between Env-YFP decreased from 166 Å (SD 90 Å) to 86 Å (SD 32 Å). The average number of interacting Env-YFP molecules per virus was 28 with a minimum of 9 and a maximum of 46. Notably, the more Env-YFP densities were found in interaction with the cell, the less unbound Env-YFP was detected on the same virus. This strongly implies clustering of Env-YFP and its cellular receptor at the interaction site. Receptor clustering at virus interaction sites had been reported before based on fluorescence microscopy studies (Lehmann et al., 2005). Yet, we did not find any indication for lattice formation (in this limited dataset), as the distribution of the distances to the nearest neighbor showed a wide plateau between 50 and 100 Å.

In the sub-volume averaging map for Env-YFP in contact with the target membrane, the membrane densities appeared less clear when the averaging was focused on the protein itself. This was particularly so for the cell membrane as compared to areas of no virus-cell interaction (Fig. 4C). We also performed averaging with the mask including either the virus or host membrane. In both cases the membrane-to-membrane distances where consistent (Fig. 4C) and the cell membrane densities did not present a clear bilayer. Noteworthy, the distance between the viral and cellular membrane (∼120 Å) is clearly larger than the height of the unbound Env-YFP trimer (∼85 Å) (Fig. 4D). As the cellular receptor is an integral multi-TM-spanning protein without significant extracellular domains, densities in these additional 35 Å must be of Env-YFP. This implies quite substantial conformational changes. Strikingly, the EM density connecting the virus to the cell appeared asymmetric (Fig. 4E) seemingly just emanating from a single protomer. The Env-YFP connection to the cell membrane is rather wide and allows for accommodation of one RBD (Fig. 4F). In contrast the connection to the virus membrane is less clear likely because the map is derived from quite a limited number of particles (332).

## 3 Discussion

Although tremendous efforts are being put into its characterization, we still lack a comprehensive understanding of the mechanistic details of retrovirus fusion. In particular, structural insights into the rearrangements that Env undergoes on the virus surface during the attachment and fusion process are scarce. Here, we analyzed the structure of free and cell bound MLV Env *in situ* in its native environment by cET and sub-volume averaging, to capture a glimpse of the molecular rearrangement of Env during the early stages of host cell interaction.

Previous reconstructions of non-interacting MLV Env report a trimer with a central cave (Wu et al., 2008) that is anchored with 3 feet in the membrane (Förster et al., 2005). The approximate height of the trimer is given with 90–100 Å. These reported overall features of Env are consistent with our reconstructions, although the height we found here is slightly lower (∼85 Å). However, the height difference might, at least in part, be due to different strength of the membrane signal and different thresholding of the EM density maps. The step-shaped overall feature of the protomers can also be found in the single particle based reconstruction (Wu et al., 2008). Apart from that, the shapes of the reconstructions – albeit having similar resolutions – are quite divergent (Supplementary Fig. 6). This might be due to differences in the virus/particle purification procedures or the reconstruction approach. Also, an effect of the production cell line or the employed virus strain cannot be excluded.

The shape of the central trimer is highly consistent between the reconstructions of the Env-YFP and Env datasets. This implies that the used production system as well as the insertion of YFP have no effect on the basic shape of the trimer at the given resolution. This only allows for YFP to be located outside the central trimer, as the central cavity of the trimer – apart from most likely containing parts of the TM subunits – is not spacious enough to accommodate the YFP densities. The biological data of viruses bearing insertions within the PRR already hinted at this, as wild type like virus growth curves (Kayman et al., 1999a, Kayman et al., 1999b, Sherer et al., 2003) imply that there is no disturbance of the Env trimer core and that YFP does not impair the functional interactions and conformational changes in the course of fusion. Indeed, weak, extended densities located more distal from the trimer core can be found in both Env-YFP reconstructions (Supplementary Fig. 1). These densities do not resemble the barrel shape of the YFP structure, but due to the flexibility of the PRR (the only position in Env where fluorescent proteins have been successfully inserted) and the hence expected flexibility in the YFP orientation we propose that they can be interpreted as a probability cloud for the localization of YFP.

Backplotting of the final EM density maps onto the original data according to the positions and orientations for the individual Env particles derived by sub-volume averaging did not reveal a particular relative arrangement of Env/Env-YFP molecules on the VLP or virus envelope. Occasionally, rows of similarly arranged trimers were present, but this might just be mere coincidence. However, backplotting did reveal good overlap with the actual present densities within the tomograms, which can serve as an additional quality control for our reconstructions.

Docking of the crystal structure of the RBD did not reveal a single best fit due to resolution limitations. However, when checking the arrangement in the trimer, it became apparent that only one fit allowed for close proximity of receptor interacting residues D86 (Bae et al., 1997, Davey et al., 1999, MacKrell et al., 1996) and W144 (Zavorotinskaya and Albritton, 1999). As the density accommodating D86 is (at the chosen threshold) only clearly present in the Env-all-protomer reconstruction, it is possible that the aa chain containing D86 allows for some movement in the molecule. Such ‘breathing’ has been described earlier for HIV Env to be possibly advantageous for establishing receptor contact (Caffrey, 2011). Among all the fits returned, there was none that resembled the one previously proposed for the inactive state (Wu et al., 2008). Interestingly, the fit of the intermediate activated state proposed in the same publication is closely similar to our fit III in Fig. 4. This might indicate that Env accommodates a different state on virions than after detergent extraction and purification.

Next, we analyzed MLV bound to its target cell. In contrast to a previously published analysis of HIV interaction with T-cells, we did not observe the described ‘entry claw’ consisting of 5–7 Env trimers associated with the cell in a ∼400 Å wide neck shape (Sougrat et al., 2007). Rather, a large interface with the cell, on average 28 connecting densities, as well as the induction of plasma membrane curvature seemingly imposed by the virus surface curvature, were observed for MLV. These differences to HIV might indicate different strategies of these viruses to enter their host cells. Other causes might be (i) that mCAT was overexpressed in our system, and hence there might be the possibility that an abundance of available receptors leads to the observed phenotype or (ii), that the sample preparation procedure including fixation and staining in the HIV study might have some effect too.

In contrast to the unbound Env-YFP trimer, the reconstruction of the bound molecule did not exhibit any discernable symmetry. Rather, the connecting density seems to emanate from a single protomer. This, together with the elongation of the structure by roughly 35 Å, implies massive structural rearrangements of the protein upon interaction with the cell. This is in contrast to observations made on HIV, as the gap between virus and host cell membrane is supposed to decrease with advances in binding (reviewed in (Blumenthal et al., 2012)). This is clearly not the case here. Due to the low numbers of events and therefore limited resolution of the retrieved average, it is not possible to identify confidently any individual Env-YFP domains. Dissociation of the Env SUs upon receptor binding has been described for HIV (reviewed in (Blumenthal et al., 2012)). Together with observed loss in Env symmetry upon binding, it is tempting to speculate that our structure might represent three TM domains – elongated in the pre-hairpin conformation – and one SU domain linking to the host cell membrane. However, higher resolution reconstructions will be needed to verify this.

Compared to unbound viruses, a substantial decrease in inter-Env distances was observed at sites of virus-cell interaction. This indicates clustering of Env at these sites, which also implies a certain mobility of Env in the viral envelope. As the fusion pore required for release of the nucleocapsid into the cytosol presumably has to be quite wide, an accumulation of fusion proteins on a fairly wide cellular surface area might support this process. Yet, as the cell line used overexpresses mCAT, it is also possible that this observation is in part caused by the abundance of available receptor molecules.

Apart from its asymmetric shape, the Env-YFP EM density map never showed a clearly defined bilayer for the cellular membrane patch being in contact with Env-YFP, even if we just used the cell membrane densities for alignment. This was surprising, as the reconstruction of the parts of the cell membrane that were not in contact with the virus did show a clearly defined bilayer. The same was observed, although to a much lesser extent, for the virus envelope. Changes in the cellular membrane due to insertion of the fusion peptide at an oblique angle have been reported amongst others for HIV (reviewed in (Apellániz et al., 2014)). Therefore, we assume that the interaction of Env-YFP with the receptor and/or the insertion of the fusion peptide into the cell membrane result in detectable loss of order in the bilayer due to lipid rearrangement or the application of mechanical stress.

With the presented data we are able to provide a glimpse of the molecular rearrangements taking place during virus-cell-interaction. Still, lots of further snapshots are missing to really be able to generate a step-by-step guide comprehensively describing the dynamics in the course of retrovirus fusion. This clearly warrants further research to improve resolution and accuracy, by acquisition of bigger datasets and analysis of additional binding stages.

## 4 Materials & Methods

### 4.1 Cell culture

Cells were kept at 37 °C/5% CO_2_ in DMEM containing 10% FBS and GlutaMax. For EM analysis, cells were seeded on C-flat gold grids (Protochips) in flat bottom μ 2x9well slides (ibidi) previously coated with poly-l-lysine (Sigma-Aldrich) 24 h before incubation with virus and subsequent plunging.

### 4.2 Virus/VLP generation

Plasmids encoding virus-derived sequences (gagpol, gagCFP, Env, EnvYFP and LTR-GFP) were transfected into COS1 cells using Fugene Xtreme Gene (Roche) according to the manufacturer once the cells had reached ∼80% confluency. Plasmids for VLP generation were transfected at the following ratios: 3/12 gagpol, 1/12 gagCFP, 4/12 Env or Env-YFP, 4/12 LTR-GFP. 48 h after transfection, the supernatant was harvested and precleared for 5 min at 4000*g*. Afterwards, the VLPs were pelleted through a 15% w/v Sucrose in PBS cushion (volume 1/6 of the supernatant) at 100,000*g* for 1 h. The pellets were left overnight at 4 °C, covered with an appropriate volume of full medium.

In the case of virus harboring DFJ8 cells, cells were grown for 72 h before the supernatant was harvested and processed as above.

### 4.3 Data acquisition

Tomograms were acquired on a FEI Tecnai F30 Polara microscope, equipped with a QUANTUM 964 postcolumn energy filter (Gatan) and a K2 Summit direct detector (Gatan) at 95,000× magnification (2.3 Å pixel size). During the acquisition, a 70 μm objective aperture (C2) and a 20 eV slit were in place. Tilt angle range was −45° to 45°, with an angular increment of 3°. The total electron dose/tomogram ranged between 40 and 60 e^−^/Å^2^ and the defocus was varied between tomograms from −1.5 to −5 μm. 0.2-0.3 s frames were collected for each image in counting mode.

### 4.4 Data processing

Frames generated during tomogram acquisition were aligned using motionCorr (Li et al., 2013) and tomograms were subsequently reconstructed with the IMOD package, (Kremer et al., 1996) using weighted back projection (Sandberg et al., 2003). Only tomograms with residual errors less than 0.5 were used for particle picking. CTF determination and correction (phase flipping only) were performed in TomoCTF (Fernández et al., 2006).

Particles were picked on 4× binned tomograms with a Gaussian filter applied, using the 3dmod program within the IMOD package (Kremer et al., 1996).

### 4.5 Sub-volume averaging

The PEET 1.9 (Nicastro et al., 2006) package was used for sub-volume averaging. An initial motive list for particle orientation was generated by calculating the vector between the virus particle center and the respective Env molecule on the virus surface. The whole dataset was employed in several runs, successively increasing spatial frequency and sampling size. Once threefold symmetry was apparent, it was applied to the whole dataset. Clean-up of particles according to the angular deviation from the initial vector connecting the VLP center with the Env molecule on its surface, as well as removal of overlapping particles, was performed after a final run. The cleaned dataset was then split, and after generation of a new initial motive list as stated above, employed for gold standard FSC calculation (cutoff 0.143) as well as the generation of the final reconstruction. Visualization of the reconstructed volumes, as well as docking of crystal structures was done in UCSF Chimera (Pettersen et al., 2004). Isosurface representation was thresholded based on the corresponding volume of the molecular weight of 3 Env SUs and the parts of 3 Env TMs units that are distal to the virus membrane (Fischer et al., 2004) for the Env trimer. For each protomer, the threshold was based on the molecular weight of 1 Env SU, and for the interacting Env, it was based on the molecular weight of 1 Env SU and the parts of 3 Env TMs units that are distal to the virus membrane. Threshold based segmentation of the final EM density maps for visualization of TM was done in Amira.

### 4.6 Data deposition/accession codes

The reconstructions and the coordinates of the fit have been deposited in the Electron Microscopy Data Bank (EMDB) at PDBe (http://www.ebi.ac.uk/pdbe/emdb/) as EMD_3357, _3363, _3365, _3373.

## Figures and Tables

**Supplementary Fig. 3:**
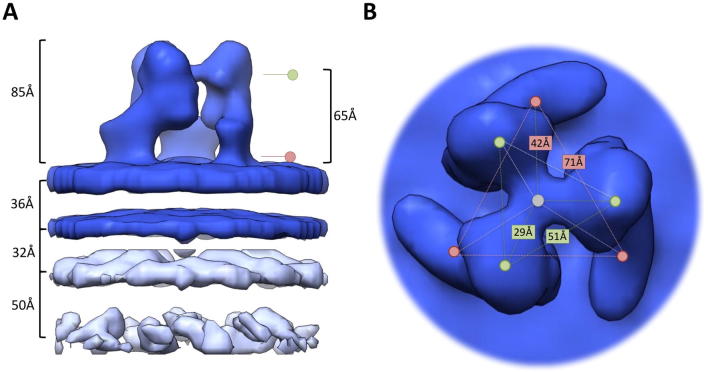


**Supplementary Fig. 6:**